# Carcinome du tube collecteur de Bellini: une nouvelle observation avec revue de la littérature

**DOI:** 10.11604/pamj.2017.27.166.9271

**Published:** 2017-07-04

**Authors:** Abdessamad El Bahri, Jaouad Chafiki, Nabil Louardi, Omar Jendouzi, Abdellatif Janane, Ahmed Ameur, Mohammed Abbar

**Affiliations:** 1 Service d’Urologie, Hôpital Militaire d’Instruction Mohammed V, Rabat, Maroc

**Keywords:** Rein, cancer, tubes collecteurs, Kidney, cancer, collecting ducts

## Abstract

Le carcinome du tube collecteur de Bellini est un type très rare des carcinomes à cellules rénales (CCR), sa fréquence est inférieure à 1%. Il dérive de la partie distale du néphron, plus précisément du tube collecteur. Ses aspects morphologiques sont extrêmement variables, rendant son diagnostic difficile. Nous rapportons le cas d'un patient âgé de 62 ans admis pour une tuméfaction non douloureuse du flanc gauche, d’apparition progressive. La tomodensitométrie a objectivé une énorme masse, occupant la partie supérieure du rein gauche. Le malade avait bénéficié d’une néphrectomie élargie. L’examen anatomo-pathologique avait objectivé un carcinome des tubes collecteurs du rein. L’évolution est exceptionnellement favorable: pas de récidives, pas de métastases locorégionales et pas de métastases à distance.

## Abstract (en)

Bellini duct carcinoma is a very rare type of renal cell carcinoma (RCC), accounting for less than 1%. It arises from the distal nephron, more specifically from the collecting duct. Its morphological features are extremely variable, making its diagnosis difficult. We report the case of a 62-year old patient admitted with a painless progressive left flank swelling. CT scan showed a huge mass occupying the upper portion of the left kidney. The patient underwent radical nephrectomy. Anatomopathological examination showed collecting duct carcinoma of the kidney. Patient’s evolution was exceptionally favorable: no recurrence, no locoregional metastasis and no distant metastasis.

## Introduction

Le carcinome des tubes collecteurs de Bellini est une forme rare (fréquence inférieure à 1%) de carcinome à cellule rénale (CCR) [1] et reste relativement méconnu par les urologues.Seul le diagnostic précoce semble offrir une réelle amélioration de survie. A partir d’un patient porteur d’un carcinome du rein ayant pour origine l’épithélium du tube collecteur (tube collecteur de Bellini), nous effectuons une revue de la littérature concernant ce type particulier de tumeur pour essayer de préciser certaines particularités diagnostiques, thérapeutiques et pronostiques.

## Patient et observation

**Figure 1:**
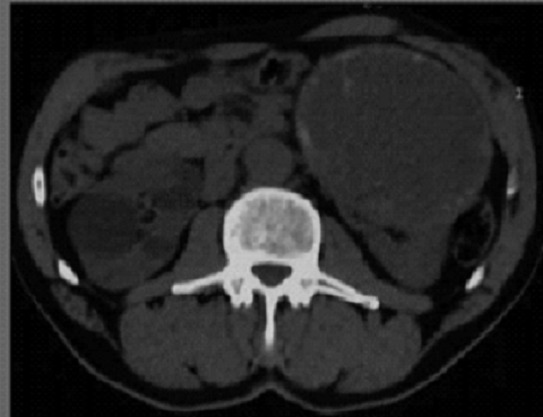
Scanner abdominal: énorme masse, occupant la partie supérieure du rein gauche

**Figure 2:**
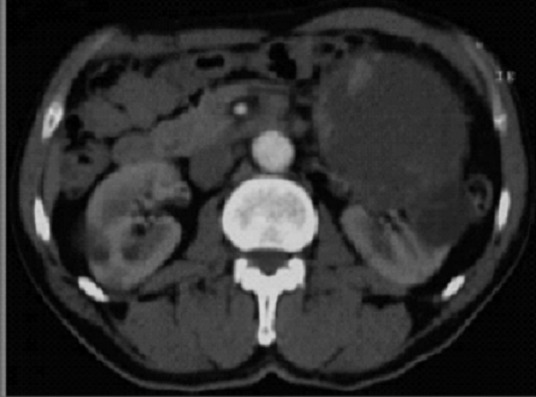
TDM abdominale, kyste à paroi calcifiée, bourgeons intra kystiques etkystes simples des deux reins

**Figure 3:**
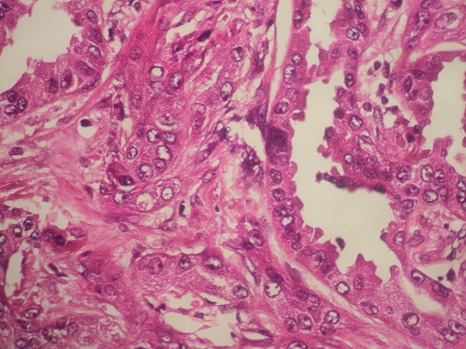
Pièce de néphrectomie élargie, masse au dépend du pôle supérieur du rein gauche

**Figure 4:**
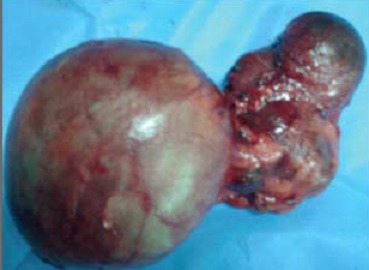
Carcinome des tubes collecteurs (Bellini): faible grossissement: prolifération carcinomateuse faite de tubes de taille variable parfois adossés au seind’un stroma desmoplasique (Gx100)

**Figure 5:**
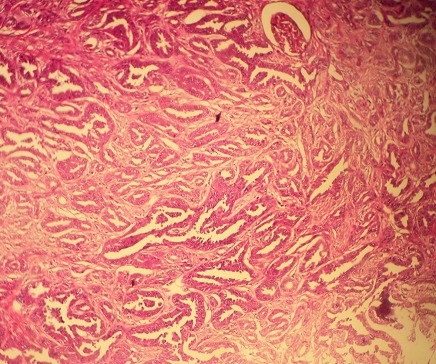
Carcinome de Bellini: fort grossissement: cellules tumorales qui tapissent les tubes carcinomateux montrant des atypies cytonucléaires modérées parfois marquées, fortement nucléolées de haut grade de Fuhrman (Gx200)

Monsieur B.H. âgé de 62ans, tabagique, a consulté en novembre 2014 pour tuméfaction non douloureuse du flanc gauche, d’apparition progressive. Le début de la symptomatologie remontait à 4 ans plus tôt par l’apparition progressive d’une douleur lombaire sans fièvre ni hématurie, le tout évoluant dans un contexte de conservation de l’état général. Aucun autre signe urinaire n’a été révélé. L’examen clinique avait objectivé un contact lombaire avec flanc gauche tendu, non douloureux à la palpation. Le reste de l’examen était par ailleurs normal. L’échographie avait objectivé une masse rétro péritonéale gauche, refoulant la rate, sans adénopathies associées. La sérologie hydatique était négative, et la fonction rénale normale. Le scanner montrait une énorme masse, occupant la partie supérieure du rein gauche, prenant le contraste de façon hétérogène, et avait mis en évidence un kyste de 12cm avec paroi calcifiée (Figure 1), des bourgeons tissulaires intra kystiques du rein gauche (Kyste stade IV de Bosniak), et des Kystes corticaux des deux reins d’aspect simple (Figure 2). Opéré par une lombotomie, le malade avait bénéficié d’une néphrectomie élargie (Figure 3), les suites ont été simples avec un recul de 3 ans. L’examen anatomopathologique a retrouvé une prolifération carcinomateuse d’architecture complexe (Figure 4 et Figure 5). Cette prolifération était faite de travées et de cordons de cellules pourvues d’un noyau rond, muni d’un nucléole bien visible ; et d’un cytoplasme éosinophile.

On avait noté également la présence de structures tubulaires. Ailleurs, la tumeur formait des papilles de petites tailles. Ses structures tumorales évoluaient au sein d’un stroma fibreux, desmoplastique (Figure 4), dont l’abondance variait d’une zone à l’autre. Ce stroma était ponctué d’éléments inflammatoires polymorphes. La tumeur infiltrait le parenchyme rénal et la graisse péri-rénale. Au sein du tissu adipeux péri-rénal existait une veine envahie par le processus tumoral avec présence d’une thrombose veineuse. Le tissu cellulo-adipeux et les structures vasculaires du hile étaient indemnes En étude immuno-histochimique, les cellules tumorales n’exprimaient pas les antigènes suivants: cytokératine7, cytokératine 20, CD10, antigène spécifique de la prostate, antigène antihépatocyte1. Elles exprimaient l’antigène épithélial membranaire (EMA). En conclusion, l’examen anathomo-pathologique avait objectivé un carcinome des tubes collecteurs du rein, de 30X20mm, grade 3 de Führman; avec extension à la graisse péri-rénale et présence de métastases ganglionnaires (T3, N2, Mx); associé à des kystes simples. L’évolution est exceptionnellement favorable: pas de récidives, pas de métastases locorégionales et pas de métastases à distance.

## Discussion

Le carcinome des tubes collecteurs de Bellini (CB) est une tumeur rénale rareet représente environ 1% des cancers épithéliaux. Cette entité a été rapportée pour la première fois en 1976 [2] par Mancilla Jimenez et a été adopté par l’OMS en 1981. Depuis, des cas épars ont été publiés. Le CB survient dans la même tranche d’âge que celle des autres CCR [3]. La majorité des patients concernés sont entre la 6^ème^ et 7^ème^ décade de leur vie comme rapporté dans notre cas (patient âgé de 62ans). Dans la littérature, 4 cas ont été décrits chez les adolescents [4]. Il existe une prédominance masculine avec sex-ratio de 2/1 [5]. Il n’existe pas de prédominance de côté [5] et aucune atteinte bilatérale synchrone ou métachrone n’a été rapportée. La moitié des patients rapportés par Chao [3], avaient une histoire familiale de cancer. Chez notre patient, aucune notion d’antécédents familiaux de cancer n’a été rapportée. Bear rapporte un cas de CB associé à un carcinome à cellules claires sur le rein controlatéral [6]. L’insuffisance rénale chronique, facteur de risque actuellement admis du carcinome rénal [7], semble moins nette pour le carcinome de Bellini, un seul cas ayant été décrit chez l’hémodialysé [8]. Le rôle du tabac dans la genèse du CB est inconnue, cependant une intoxication tabagique est retrouvée dans 41% des cas dans la série de Dimopoulos et al [4] et retrouvée chez notre patient également.La plupart des CCR sont de découverte fortuite. A l’opposé, le carcinome de Bellini est le plus souvent symptomatique [3]. Il s’agit le plus souvent (50-66,7%) d’une hématurie macroscopique, de lombalgies (40%) et parfois d´une masse palpable de la fosse lombaire ou du flanc [1]. Chez notre patient le mode de révélation était une tuméfaction non douloureuse du flanc gauche. Parfois le diagnostic est posé à un stade évolué de la maladie devant une altération de l’état générale avec amaigrissement et métastases ganglionnaires ou viscérales d´emblée (26%). La présence d´un thrombus cave est rapporté dans 2 cas [9]. Alors que dans 8% des cas La découverte est fortuite radiologique [10]. Sur le plan Radiologique, le CB n ´a rien de spécifique.

Toutefois pour FUKUYA, en cas de faible volume, la tumeur se développe vers le sinus du rein sans en déformer les contours externes et se rehausse faiblement après injection de produit de contraste [10]. Le diagnostic de CB est anatomopathologique, macroscopiquement, Le carcinome de Bellini est le plus souvent situé dans la région centrale du rein, en plein parenchyme il s’agit d’une tumeur de grande taille. Elle est ferme, jaune ou grisâtre à la coupe, en général mal limitée [6, 9] avec souvent des nodules satellites et des remaniements hémorragiques. L’analyse Microscopique trouve une tumeur glandulaire constituée de tubes à contours irréguliers et de topographie médullaire. Il s’agit le plus souvent de volumineuses cellules à cytoplasme éosinophile avec des noyaux de grande taille et fortement nucléolés, de grade nucléaire élevé [9]. Pour FONDIMARE, l’aspect macroscopique associé à une architecture tubulaire, microkystique et papillaire, ainsi que l’aspect cytologique seraient fortement évocateurs du diagnostic [9]. Il peut exister aussi des aspects sarcomatoides focalisés [6]. L’existence de lésions dysplasiques des tubes collecteurs au voisinage de la tumeur est aussi un élément du diagnostic [11]. La cytogénétique devrait permettre de mieux connaître les évènements moléculaires au cours du CB. Contrairement à l´adénocarcinome rénal où les aberrations chromosomiques concernent surtout les chromosomes 3, 7 et Y, SCHOENBERG retrouve dans 50% une perte d´hétérozygotie au niveau des chromosomes 8p et 13q sur une série de 6 CB [12]. Les travaux récents portent sur l’évaluation des facteurs pronostiques prédictifs plus spécifiques à ce type histologique afin d’améliorer la prise en charge de ces patients [13]. La néphrectomie totale a sa place dans la stratégie thérapeutique des patients, même métastatiques. Une chimiothérapie adjuvante par association gemcitabine-sels de platine, également appliquée dans les carcinomes urothéliaux, peut être proposée en attendant un meilleur recul de l’apport des thérapies ciblées [14].

## Conclusion

Le carcinome de Bellini est une tumeur maligne rénale rare, de mauvais pronostic en raison de la fréquence de sa découverte au stade métastatique. Le diagnostic histologique, est principalement basé sur l'analyse immunohistochimique. Cette tumeur a bénéficié de l’apport de la thérapie ciblée. En revanche, vu la rareté de notre entité histologique, Le traitement standard reste toujours la NTE, en attendant la validation des autres modalités thérapeutiques.

## Conflits d’intérêts

Les auteurs ne déclarent aucun conflit d'intérêt.
